# Comorbidity in Adults with Epilepsy — United States, 2010

**Published:** 2013-11-01

**Authors:** Norbert T. Kadima, Rosemarie Kobau, Matthew M. Zack, Sandra Helmers

**Affiliations:** SciMetrika, Research Triangle Park, North Carolina; Div of Population Health, National Center for Chronic Disease Prevention and Health Promotion, CDC

Epilepsy, a spectrum disorder characterized by recurring seizures, affects approximately 2.3 million U.S. adults (1,2). Epilepsy poses challenges because of uncontrolled seizures, treatment complexity, social disadvantages (e.g., unemployment), and stigma (2,3). Persons with epilepsy are at increased risk for early mortality and for comorbidities that can complicate epilepsy management, increase health-care costs, and shorten the lifespan (2,4–7). Numerous studies have described higher rates of psychiatric comorbidity (e.g., depression and anxiety) in persons with epilepsy (2,7).* However, fewer studies have examined nonpsychiatric comorbidity in a nationally representative U.S. sample of adults with epilepsy. To assess the prevalence of nonpsychiatric comorbidities, CDC analyzed data from the 2010 National Health Interview Survey (NHIS). Adults with epilepsy had a higher prevalence of cardiovascular, respiratory, some inflammatory, and other disorders (e.g., headache, migraine, and various other types of pain) than adults without epilepsy. Public health agencies can work with health-care providers, the Epilepsy Foundation, and other partners to ensure that adults with epilepsy have access to health promotion resources and chronic disease self-management programs.

CDC analyzed data from adults aged ≥18 years who responded to NHIS, an annual cross-sectional survey of the civilian, non-institutionalized U.S. population.† The NHIS Sample Adult component included questions that determined epilepsy status. Adults who responded “yes” to ever having been told by a doctor or other health professional that they had a seizure disorder or epilepsy were considered as having “any epilepsy.” Those with any epilepsy who either were currently taking medication to control it, had one or more seizures in the past year, or both were classified as having “active epilepsy” (1). Those with any epilepsy who were neither taking medication for epilepsy nor had a seizure in the past year were classified as having “inactive epilepsy” (1).§ All remaining adults were classified as having “no history of epilepsy.” These case-ascertainment questions and case definitions meet standards for epidemiologic studies of epilepsy, including having acceptable positive predictive values for identifying clinical cases of epilepsy (1).

Nonpsychiatric conditions that were selected included some shown to be previously associated with epilepsy, and others not widely examined, but of interest to epilepsy providers (e.g., any liver condition). Statistical software was used to account for the complex NHIS survey design. Percentage estimates were age-adjusted to the 2000 U.S. Census population to account for age as a confounder and to facilitate comparisons.¶ Estimates were considered reliable if their relative standard errors were <30% and differences were considered statistically significant if their 95% confidence intervals did not overlap. All reported differences are statistically significant. The 2010 NHIS Sample Adult Component conditional response rate was 77.3%, and the final response rate was 60.8%.

Cardiovascular and metabolic disorders and their associated risk factors were common among adults with epilepsy (Table). The age-adjusted prevalence of any heart disease was higher among adults with any epilepsy (18.3%), including both active epilepsy (19.5%) and inactive epilepsy (16.7%), than among those without epilepsy (11.3%). Adults with any epilepsy were more likely to have been told they had high blood pressure (34.2%) than those without epilepsy (29.0%). More adults across all epilepsy groups (range: 8.8%–18.3%) had experienced a stroke than adults without epilepsy (2.4%). More adults with any epilepsy (7.1%) were told they had prediabetes than adults without epilepsy (4.3%). Adults with any epilepsy (34.1%) and inactive epilepsy (40.3%) were more likely to be obese than adults without epilepsy (27.5%).

Considering respiratory disorders, more adults with any epilepsy (5.5%) and active epilepsy (6.2%) had emphysema than those without the disorder (1.7%). More adults with any epilepsy (7.5%) and active epilepsy (8.5%) had chronic bronchitis in the past year than adults without epilepsy (4.1%). More adults with any epilepsy (19.2%) and inactive epilepsy (19.9%) had asthma than those without epilepsy (12.6%). However, adults with active epilepsy were more likely to have had an asthma attack in the past year (51.9%) than adults without epilepsy (32.6%).

Some disorders that can be caused or mediated by inflammation also were more common in adults with epilepsy. For example, significantly more adults across all epilepsy groups than adults without epilepsy had a history of dermatitis, arthritis, recent joint pain, and other types of pain including, neck, facial, and low back pain. Across all groups, more than twice as many adults with epilepsy than adults without epilepsy had experienced recent, severe headache or migraine.

Cancer was more common in adults with any epilepsy (11.3%) than adults without epilepsy (8.1%). Adults with any epilepsy were more likely to have had peptic ulcer disease and to have had ulcer symptoms in the past year than adults without epilepsy. More adults with any epilepsy had a liver condition than those without epilepsy. In addition, adults with epilepsy, especially active epilepsy, were more likely to report four or more medical comorbidities and less likely to report no other comorbidities than adults without epilepsy (Figure).

## Editorial Note

In this study, many U.S. adults with epilepsy (especially those with active epilepsy) reported cardiovascular, respiratory, and other disorders and various types of pain, consistent with other U.S. and international reports (2,4,6). These disorders might result from shared disease mechanisms (e.g., migraine or stroke), social disadvantages associated with chronic disease (e.g., risk-factor clustering), treatment side effects (e.g., weight gain), or shared genetic, environmental, or other factors (2,5,8). Adults with epilepsy also report higher rates of smoking and physical inactivity (3), which increase risk for heart disease and respiratory disorders.

This study aligns with the U.S. Department of Health and Human Services Initiative on Multiple Chronic Conditions by identifying the burden of co-occurring conditions in adults with epilepsy to foster more encompassing approaches to address this burden (9). Although controlling seizures is a priority for epilepsy care, preventing, limiting, and reversing associated comorbidity remains critical to improving health and quality of life (2,10). The added challenges of managing multiple comorbidities among adults with epilepsy can further threaten their well-being and ability to function optimally (5). Thus, improved awareness and understanding among neurologists and primary-care providers regarding the common medical comorbidities reported with epilepsy along with better screening, diagnosis, and treatment of comorbidity in persons with epilepsy are necessary. A previous study found that most adults with epilepsy visited a general doctor in the preceding year, but only about one third saw a neurologist or epilepsy specialist (1). The extent to which evidence-based practice guidelines are used remains unclear (2), and health-care providers might focus only on one condition, ignoring care coordination (9). Managing comorbidity requires that primary and specialty-care providers work together to help patients with epilepsy manage both their epilepsy and other disorders, using appropriate clinical guidelines (2).

What is already known on this topic?

Persons with epilepsy might be at increased risk for some mental and physical disorders.

What is added by this report?

This study, based on the 2010 National Health Interview Survey, found that adults with epilepsy reported co-occurring cardiovascular, respiratory, some inflammatory, and other disorders more frequently than respondents without epilepsy.

What are the implications for public health practice?

Epidemiologic studies to show how epilepsy is related to these comorbid conditions could help identify preventable risk factors. Greater collaboration among public health agencies, health-care providers, the Epilepsy Foundation, and other partners might ensure that adults with epilepsy have access to chronic disease self-management programs and to general disease prevention and health promotion information and services.

The findings in this report are subject to at least eight limitations. First, because the estimates rely on self-reported data, they might be subject to reporting bias; however, comparable findings in other population surveys suggest bias is minimal (3). Second, the reported cases of epilepsy are not classified by seizure type, severity, or etiology. Third, certain acute seizures or nonepileptic seizures might have been misclassified as epilepsy, thus overestimating prevalence. However, significant skewing of results is unlikely because of the low incidence of nonepileptic seizures in the general population (1,3). Fourth, epilepsy prevalence might be underestimated because of underreporting associated with repercussions from disclosing epilepsy (1,3) and the exclusion of institutionalized adults from NHIS. Fifth, because the onset of epilepsy relative to that of the other co-occurring disorders is unknown, inferring causation or overlap between these disorders is difficult. Sixth, small sample sizes limited comparisons. Seventh, the low response rate could have understated or overstated these associations. Finally, because of different study methodologies, estimates of comorbidities differ from those in a previous report.**

Ensuring that adults with epilepsy are screened for common risk factors might help prevent onset of co-occurring disorders that can worsen quality of life over time. Preventing stroke, a common risk factor for epilepsy in adults, also might minimize epilepsy incidence in those at higher risk (e.g., adults who have experienced prior head trauma) (2). Future studies can look at mechanisms that relate epilepsy to these comorbid conditions. Evidence-based programs that can help adults with epilepsy learn effective self-management skills (e.g., medication adherence and emotional management) are available.†† Greater collaboration among public health agencies, health-care providers, local Epilepsy Foundation affiliates, and other community epilepsy groups might ensure that adults with epilepsy have access to chronic disease self-management programs and to health promotion resources (e.g., smoking cessation programs and interventions to reduce obesity).

## Figures and Tables

**FIGURE:**
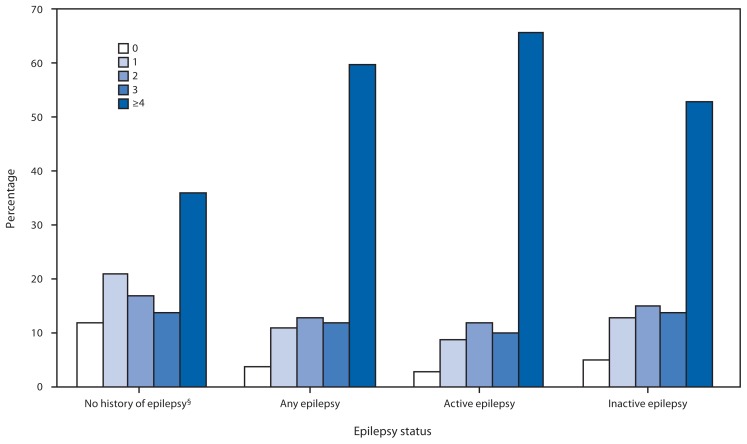
Percentage^*^ of adults with selected nonpsychiatric conditions,^†^ by number of conditions and epilepsy status — National Health Interview Survey, United States, 2010 ^*^ Unadjusted estimates. ^†^ Includes self-reported heart disease (coronary heart disease, angina pectoris, myocardial infarction or any other heart disease); high blood pressure; stroke; diabetes mellitus; prediabetes; emphysema; chronic bronchitis; asthma; hay fever; sinusitis; dermatitis/eczema; arthritis; joint pain, aching, or stiffness; neck pain; low back pain; facial or jaw pain; severe headaches or migraine; cancer; ulcer; liver condition; and overweight/obesity (body mass index ≥25). ^§^ Because of different methodologies, estimates of comorbidities among adults with no history of epilepsy differ from those in a previously published report (Ward BW, Schiller JS. Prevalence of multiple chronic conditions among US adults: estimates from the National Health Interview Survey, 2010. Prev Chronic Dis. 2013;10:E65).

**TABLE:** Percentage* of adults with selected nonpsychiatric conditions,† by epilepsy status — National Health Interview Survey, United States, 2010

	No history of epilepsy			Any epilepsy			Active epilepsy			Inactive epilepsy		
				
Condition	No.	%	(95% CI)	No.	%	(95% CI)	No.	%	(95% CI)	No.	%	(95% CI)
**Total** §	**26,659**	**98.2**	**(98.0–98.5)**	**480**	**1.8**	**(1.5–2.0)**	**277**	**1.0**	**(0.8–1.2)**	**198**	**0.8**	**(0.6–0.9)**
Any heart disease¶	3,218	11.3	(10.9–11.8)	98	18.3	(14.7–22.6)	61	19.5	(14.3–25.9)	35	16.7	(11.9–22.9)
Hypertension	8,647	29.0	(28.4–29.6)	194	34.2	(29.7–39.0)	124	34.7	(28.7–41.2)	68	32.2	(25.3–40.0)
Stroke	768	2.4	(2.2–2.7)	72	14.3	(11.1–18.2)	54	18.3	(13.4–24.4)	17	8.8	(5.6–13.6)
Diabetes mellitus	2,709	8.7	(8.2–9.1)	58	10.4	(7.7–14.0)	38	10.5	(7.3–14.9)	19	9.2	(5.3–15.4)
Prediabetes	1,071	4.3	(3.9– 4.6)	35	7.1	(4.7–10.4)	—	—**	—**	—	—	—
Normal/underweight	9,514	38.1	(37.3–38.9)	144	32.8	(27.5–38.6)	83	34.7	(27.0–43.3)	59	32.5	(25.5–40.4)
Overweight	8,938	34.5	(33.7–35.2)	153	33.1	(28.4–38.2)	94	37.1	(30.0–44.8)	57	27.2	(20.8–34.6)
Obese	7,247	27.5	(26.7–28.2)	163	34.1	(28.9–39.8)	89	28.2	(21.8–35.5)	74	40.3	(32.0–48.2)
Emphysema	500	1.7	(1.5–2.0)	30	5.5	(3.5–8.3)	20	6.2	(3.7–10.0)	—	—	—
Chronic bronchitis	1,171	4.1	(3.8–4.5)	42	7.5	(5.2–10.6)	27	8.5	(5.2–13.3)	—	—	—
Asthma	3,243	12.6	(12.0–13.2)	104	19.2	(15.2–24.0)	58	17.0	(12.3–23.0)	43	19.9	(14.1–27.4)
Current asthma	2,142	65.0	(62.8–67.0)	74	59.8	(47.2–71.1)	45	67.4	(48.4–82.0)	26	55.1	(38.8–70.4)
Asthma attack in past 12 mos	1,106	32.6	(30.7–34.6)	49	36.6	(25.9–48.7)	32	51.9	(34.8–68.6)	15	28.7	(18.8–41.3)
Hay fever	1,975	7.6	(7.1–8.0)	47	7.5	(5.4–10.4)	29	8.1	(5.0–12.8)	18	7.1	(4.1–11.9)
Sinusitis	3,418	12.6	(12.1–13.2)	86	15.6	(12.2–19.7)	48	14.7	(10.3–20.4)	37	16.3	(11.5–22.6)
Dermatitis	2,556	10.0	(9.4–10.5)	83	17.5	(13.5–22.3)	46	15.8	(11.3–21.7)	36	19.5	(13.7–27.0)
Arthritis	6,250	21.4	(20.8–22.0)	180	30.9	(27.3–34.8)	114	31.3	(26.1–37.0)	64	29.0	(23.1–35.5)
Pain or stiffness in a joint	8,832	31.9	(31.2–32.7)	257	47.5	(42.4–52.6)	158	49.1	(41.7–56.5)	96	44.9	(37.3–52.7)
Neck pain	4,240	15.2	(14.6–15.8)	143	25.7	(21.3–30.8)	89	28.5	(22.3–35.6)	51	22.7	(16.8–29.9)
Low back pain	7,736	28.2	(27.4–29.0)	219	40.1	(35.2–45.3)	129	40.9	(33.8–48.5)	87	39.9	(32.1–48.1)
Sciatica	2,883	34.3	(33.0–35.7)	124	58.3	(49.2–66.9)	73	60.8	(48.9–71.5)	49	54.1	(41.9–65.9)
Facial ache or pain in the jaw	1,256	4.8	(4.4–5.2)	73	14.2	(10.7–18.6)	43	13.4	(9.1–19.4)	28	14.2	(9.1–21.4)
Severe headache or migraine	4,277	16.2	(15.7–16.8)	174	34.7	(30.1–39.5)	104	35.5	(28.5–43.1)	69	33.7	(26.8–41.4)
Cancer	2,269	8.1	(7.7–8.5)	62	11.3	(8.6–14.9)	41	11.2	(7.9–15.8)	20	11.5	(7.0–18.5)
Ulcer	1,798	6.2	(5.8–6.6)	69	12.4	(9.2–16.5)	38	11.5	(8.0–16.2)	28	15.2	(9.8–22.9)
Ulcer in the past 12 mos	501	28.9	(25.8–32.1)	31	47.1	(32.7–61.9)	19	45.3	(28.4–63.4)	10	45.6	(27.9–64.5)
Liver condition	404	1.3	(1.1–1.5)	18	3.0	(1.7–5.0)	—	—	—	—	—	—

**Abbreviation:** CI = confidence interval.

* Tabulated percentages were age-adjusted to 2000 U.S. Population Census estimates. The age groups used for adjustment were 18–44, 45–64, 65–74, and ≥75 years.

† Based on reporting to a doctor or other health professional or being told by a doctor or other health professional. For different conditions, the time period asked differed (e.g., condition or symptoms in past 12 months, 3 months, or 30 days). Additional information available at http://www.cdc.gov/nchs/nhis/nhis_questionnaires.htm.

§ The number of respondents is unweighted; the percentage estimates are weighted.

¶ Includes coronary heart disease, angina pectoris, heart attack, or any other heart condition or disease.

** Relative standard error exceeded 30%.

## Footnotes

* For example, the prevalence of any mental health disorder in the past 12 months was found to be 23.5% among persons with epilepsy, compared with 10.9% among those without epilepsy, and the lifetime prevalence of suicidal ideation was 25.0% in persons with epilepsy, compared with 13.3 % in those without epilepsy (7).

† Additional information available at http://www.cdc.gov/nchs/nhis.htm.

§ Five cases of epilepsy lacked information on medication usage or on seizure occurrence and could not be classified as either active or inactive.

¶ Age groupings used for age-adjustment were 18–44, 45–64, 65–74, and ≥75 years. Age-adjustment standards are available at http://seer.cancer.gov/stdpopulations/stdpop.singleages.html.

** Ward BW, Schiller JS. Prevalence of multiple chronic conditions among US adults: estimates from the National Health Interview Survey, 2010. Prev Chronic Dis 2013;10:E65.

†† Information regarding epilepsy self-management programs is available at http://www.cdc.gov/epilepsy.

## References

[1] CDC (2012). Epilepsy in adults and access to care—United States, 2010. MMWR.

[2] Institute of Medicine (2012). Epilepsy across the spectrum: promoting health and understanding.

[3] CDC (2008). Epilepsy surveillance among adults—19 states, Behavioral Risk Factor Surveillance System, 2005. MMWR.

[4] Sander JW (2013). Comorbidity and premature mortality in epilepsy. Lancet.

[5] Institute of Medicine (2012). Living well with chronic illness: a call for public health action.

[6] Ottman R, Lipton RB, Ettinger AB (2011). Comorbidities of epilepsy: results from the Epilepsy Comorbidities and Health (EPIC) survey. Epilepsia.

[7] Tellez-Zenteno JF, Patten SB, Jetté N, Williams J, Wiebe S (2007). Psychiatric comorbidity in epilepsy: a population-based analysis. Epilepsia.

[8] Schuit AJ, van Loon AJ, Tijhuis M, Ocké MC (2002). Clustering of lifestyle risk factors in a general adult population. Prev Med.

[9] Parekh AK, Goodman RA, Gordon C, Koh HK, HHS Interagency Workgroup on Multiple Chronic Conditions (2011). Managing multiple chronic conditions: a strategic framework for improving health outcomes and quality of life. Pub Health Reports.

[10] National Institute of Neurological Disorders and Stroke (2013). 2007 epilepsy research benchmarks.

